# Qualitative assessment of nasopharyngeal aspirates as an alternative to tracheal aspirates in extremely preterm infants

**DOI:** 10.3389/fped.2026.1804309

**Published:** 2026-06-10

**Authors:** Fernando Garrido-Muñoz, Alejandro Fernández-Vega, Rebeca Gregorio-Hernández, Alberto Trujillo-Fagundo, Almudena Alonso-Ojembarrena

**Affiliations:** 1 Instituto de Biomedicina de Sevilla (IBiS), Hospital Universitario Virgen del Rocío/CSIC/Universidad de Sevilla, Seville, Spain; 2 Neonatology Department, Gregorio Marañon University Hospital, Madrid, Spain; 3 Neonatal Intensive Care Unit, Dr. Josep Trueta University Hospital, Girona, Spain; 4 Neonatal Intensive Care Unit, Puerta del Mar University Hospital, Cádiz, Spain; 5 Perinatal Brain Damage Group, Biomedical Research and Innovation Institute of Cadiz (INiBICA), Puerta del Mar University Hospital, Cadiz, Spain

**Keywords:** bronchopulmonary dysplasia, computational biology, data collection, lung diseases, neonate, proteomics

## Abstract

The search for novel biomarkers for respiratory diseases, especially those associated with extreme premature birth, has been a challenge over the past several decades. In this study, we performed a qualitative comparison of proteomic profiles derived from a novel noninvasive sampling methodology, nasopharyngeal aspiration, and an established invasive methodology, tracheal aspiration, in preterm neonates, to determine whether nasopharyngeal aspirates (NPAs) are suitable for respiratory biomarker research. Paired NPA and tracheal aspirate (TA) samples (*N*=5) were collected from 1-week-old neonates and analyzed via high-throughput proteomics. The shared and specific sample proteins were studied. Enrichment analysis of the shared proteins (649) revealed 2,937 functional proteomic database hits (terms): 392 proteins (60.4%) were specific to lung cells related to preterm lung pathologies; and a number of relevant transcription factors (74) and microRNAs (90) were also found. Biological process, molecular function and cellular component terms specific to NPA and TA displayed similarities of 44%, 54% and 78%, respectively. However, NPA proteins were enriched in terms related to the epithelium, whereas TA proteins remained specific to lung tissue. This comparative study highlights the potential of NPA samples as an alternative material for lung biomarker research applied to preterm neonates suffering from respiratory pathologies.

## Introduction

The use of invasive sampling methodologies (ISMs) to gather biological sample material from extremely low-gestational-age newborns (ELGANs) has been a common practice in recent decades in neonatal intensive care units (NICUs) (1–3). This is because ISMs often offer a better tissue specificity (1, 2); for example, samples such as blood or plasma provide high resolution of global biomolecular processes and immune responses following pathology (4–6). Previous research has achieved improvements in these methodologies in terms of minimizing invasiveness (7). However, implications of these methods for preterm health, due to the stress incurred during and after sample collection, remain an issue to be assessed (8, 9).

Bronchopulmonary dysplasia (BPD) is a common pulmonary disease in ELGANs and is recognized for its multifactorial nature and its incidence in preterm neonates born before 28 weeks gestational age (GA, 30%–80%) (10). BPD pathology may be caused by factors related to the early stage of lung and alveolar tissue development, abnormal immune response and general tissue inflammation caused by oxygen toxicity, or the early use of invasive mechanical ventilation (IMV) (11–13). Long-term comorbidities, such as pulmonary hypertension (14), neurodevelopmental pathologies (2) or even death, have been associated with severe BPD (15). ISMs have been widely applied to study BPD. Bronchoalveolar lavage and tracheal aspiration are the two most commonly used ISMs in BPD studies (9). Although both offer high tissue specificity, they require endoscopic procedures, which may compromise neonatal health, especially for ELGANs (2, 9). Both of these methods are used to obtain samples from previously intubated patients, but tracheal aspirate (TA) collection is less invasive (7). Nevertheless, the use of these ISMs for studies of BPD patients hampers patient recruitment, increasing the difficulty of research on BPD and other respiratory pathologies, particularly as the use of early invasive intubation has been significantly decreased over the past few decades in favor of noninvasive approaches (13).

Noninvasive sampling methodologies (NISMs) have emerged to improve patient wellness and, in some cases, to facilitate patient recruitment for research centered on rare diseases or specific regions that are difficult to access (16, 17). Nasopharyngeal aspiration is a novel NISM with low technical requirements that can increase patient eligibility.

High-throughput proteomics has emerged as a well-founded omics methodology applicable to BPD (4, 16–19). Proteomics relies on the study of the proteome, which is defined as the set of proteins in a biological sample (20). Recent studies on urine and oral secretions have revealed the potential of proteomic analyses of NISM samples to identify biomarkers for BPD (16, 17). In this study, we aimed to evaluate the qualitative similarities and differences of proteomic data obtained from paired nasopharyngeal aspirate (NPA) and TA samples in a cohort of 1-week-old preterm infants born before 30 weeks of gestation. In addition, we aimed to identify common lung-specific proteins from both sample types to establish NPA as a potential NISM alternative for future research on biomarkers for the early detection of respiratory diseases.

## Methods

### Study population

Patients with paired samples (NPA and TA) were extracted from the PARADYS project (Clinical Trials: NCT04785859), a multicenter next-generation study of BPD risk evaluation in infants born before 30 weeks GA. Infants from 3 Spanish NICUs between November 2020 and June 2023 were included, following the exclusion criteria described in Garrido-Muñoz et al., 2025 (21). The parents or legal guardians provided written informed consent for participation; the study was approved by each regional ethics committee (code: NEO-LUS-20_01). All samples were obtained and processed following the relevant guidelines and regulations, in accordance with the Declaration of Helsinki (22). Patients were divided into 3 groups: no-BPD, BPD [requiring supplementary oxygen up to 36 weeks GA (23)] and death before 36 weeks GA (deceased). Three objectives were set for this study: i) to study proteome similarities between paired NPA and TA samples at one week of life; ii) to identify differences between proteins exclusive to each sample type; and iii) to evaluate the potential impacts of a new NISM for the characterization of the respiratory health proteome of preterm neonates.

### Sample preparation

NPA and TA samples were collected from 1-week-old neonates by trained nurses. NPA collection consisted of the use of a perforated tube to collect any biological material produced during patient respiration, whereas TA consisted of biological material extraction after tracheal suction via an endotracheal tube. All samples were collected in 1 mL of physiological saline solution and then centrifuged for 30 min at 1.500 g and 4 °C to remove debris. The resulting supernatant was then stored in aliquots at −80 °C for proteomic processing. All aliquots were processed at a single center (Proteomics Unit, Biomedical Research and Innovation Institute of Cádiz, Cádiz, Spain). The samples were first centrifuged for 30 min at 17,000 g and 4 °C for additional debris removal, and the supernatant was subsequently transferred to new protein LoBind Eppendorf tubes (Eppendorf, MA, USA). Then, precipitation with cold acetone was carried out for each resulting aliquot. After acetone removal, the protein pellets were resuspended in 100 µL of lysis buffer containing 5% SDS at 95 °C. Protein quantification was performed in a FLUOstar Omega fluorimeter (BMG Labtech, Ortenberg, Germany) via the Pierce BCA Protein Assay Kit (Thermo Fisher Scientific, Waltham, MA, USA). Digestion of 40 µg of protein from each sample was performed via MagResin Hydroxyl beads (ReSyn Biosciences, Edenvale, Gauteng, South Africa). First, 80 µL of lysis buffer containing 5% SDS and 235 µL of acetonitrile were added to the protein extract; then, magnetic beads were introduced at a 1:4 bead-to-protein ratio (8 µL in total); after two cycles of 10 min of agitation at room temperature and two sets of three 5-minute washes with acetonitrile and ethanol, 300 µL of 50 mM ABC was added, along with digestive enzymes at a 1:50 trypsin/Lys-C-to-protein ratio (4 µL per sample; Promega Corporation, MA, USA). The samples were then incubated overnight with agitation at 37 °C. On the following day, the samples were acidified with 70 µL of 5% trifluoroacetic acid; an extract of 3 µg of protein from each sample was subjected to standard EvoTip protein purification and assessment (Evosep, Odense, Denmark). Finally, the resulting eluted purified extracts were dried (Thermo Fisher Scientific SpeedVac, 2 h, V-AQ mode), aliquoted and stored at −80 °C for further mass spectrometry analysis.

### Nano-liquid chromatography-mass spectrometry acquisition

The samples (1 µL, 100 ng of protein digested on the column) were resuspended in 60 µL of 0.1% water-formic acid solution prior to analysis on a timsTOF Pro (Bruker, Billerica, MA, USA) Q-TOF (Q: quadrupole; TOF: time-of flight) mass spectrometer coupled to a nanoElute (Bruker) liquid chromatography (LC) system. A C18 PepSep column (150 mm × 75 µm, 1. 9 µm id, 120 Å pore size; Bruker Daltonics, Bremen, Germany) was used for this experiment. The LC and gradient parameters were the same: peptide elution was performed with a 30-minute gradient from 2 to 35% B (acetonitrile-formic acid solution 0.1%), followed by 3 min to increase B from 35% to 95%, and 7 min of column cleaning (95% B). Other parameters, such as the chromatography flow rate and the column oven, were 250 nL/min and 40 °C, respectively. A capture nanoelectrospray source (Bruker) at 1,500 V was used for peptide ionization. Runs were analyzed via a diaPASEF acquisition mode, as described in Arenas-De Larriva et al. (24).

### Data processing

The results from the diaPASEF runs were analyzed with DIA-NN software (version 1.8.1) (25). Sample raw diaPASEF runs were loaded and further used to search the human SwissProt reviewed proteome in FASTA format (20,399 canonical proteins; 17 November 2022). The software parameters were set to default, with a few exceptions: the library-free search/library generation option was checked, the peptide length rate was set from 7 to 52, the precursor FDR was set to 1%, the mass accuracies (general and MS1) were set to 20 and 15 ppm, respectively, and the match-between-rounds option was selected. No additional protein-level filtering was applied, and protein groups supported by a single unique peptide were retained. The output was a protein expression matrix containing all the relative intensities of all the identified protein groups for each sample.

The resulting protein expression matrix contained a total of 3,701 protein groups, excluding contaminant and reverse identifications; it was preprocessed via RStudio (RStudio 2021.09.0, Build 351 “Ghost Orchid” for macOS). First, proteins were filtered to the most probable protein group, protein ID and gene ID; this consisted in the selection of the first reported term of the raw protein expression matrix. Subsequently, duplicated rows and rows with no protein ID were removed to ensure protein consistency, resulting in a matrix of 3,699 rows. Second, 3 independent data matrices were created, two representing each sample type independently (NPA and TA) and one for both types together. Third, rows were filtered to remove those that contained missing values (MVs); with this conservative strategy, we aimed to retain proteins with complete observations between samples. Fourth, the resulting protein matrix numerical data were transformed via log_2_ transformation for visualization purposes. Finally, the 3 matrices were NPA, TA, and common non-MV proteins (shared proteins, SP).

### Data analysis

The methods described below were performed via RStudio. Figures were generated with the ggplot2 package and further processed via Linearity curve (5.6.2, Linearity GmbH, Berlin, Germany).

The numbers of proteins in individual samples were calculated with baseR to compare the baseline protein numbers between sample types. Following the filtering strategy described above, shared and exclusive proteins were obtained using a presence/absence framework implemented via the VennDetail package (26). This approach was applied for qualitative protein assessment rather than for evaluating quantitative differences in abundance between sample types, thereby avoiding assumptions associated with missing value imputations. Heatmaps were generated with the complexHeatmap package (27). Two heatmaps were built with the top 50 exclusive proteins according to their average protein expression values in each sample type; features were clustered via the Euclidean hierarchical clustering method; and no sample clustering was performed. An additional heatmap was built with the SP matrix; the features were clustered as previously described, and the samples were clustered via Ward's distance hierarchical clustering method. To test possible correlations between paired samples, Pearson correlation studies were performed on each sample pair (Figures 2B–F), and the R values and *P* values were extracted. Functional enrichment analyses were performed with NPA- and TA-exclusive protein matrices, as well as the SP matrix; corresponding protein gene IDs from protein matrices were used to enhance database search. The package gprofiler2 (28) was used for these analyses. The enrichment terms were extracted by significance according to FDR from the available human databases (Gene Ontology (GO), Kyoto Encyclopedia of Genes and Genomes (KEGG), Reactome (REAC), Human Protein Atlas (HPA), Human Phenotype Ontology (HP), Transfac (TF), miRTarBase (MIRNA) CORUM protein complexes (CORUM) and WikiPathways (WP)). Semantic similarity analysis was applied to the GO terms [biological process (BP), molecular function (MF) and cellular component (CC)] of the NPA and TA results via the GOSemSim package (29). The similarity between the NPA- and TA-enriched terms was calculated via the method described in Wang et al. (30) and summarized via the BMA method, which consists of the average all maximum similarities between NPA and TA.

## Results

### Study population

**Figure 1:**
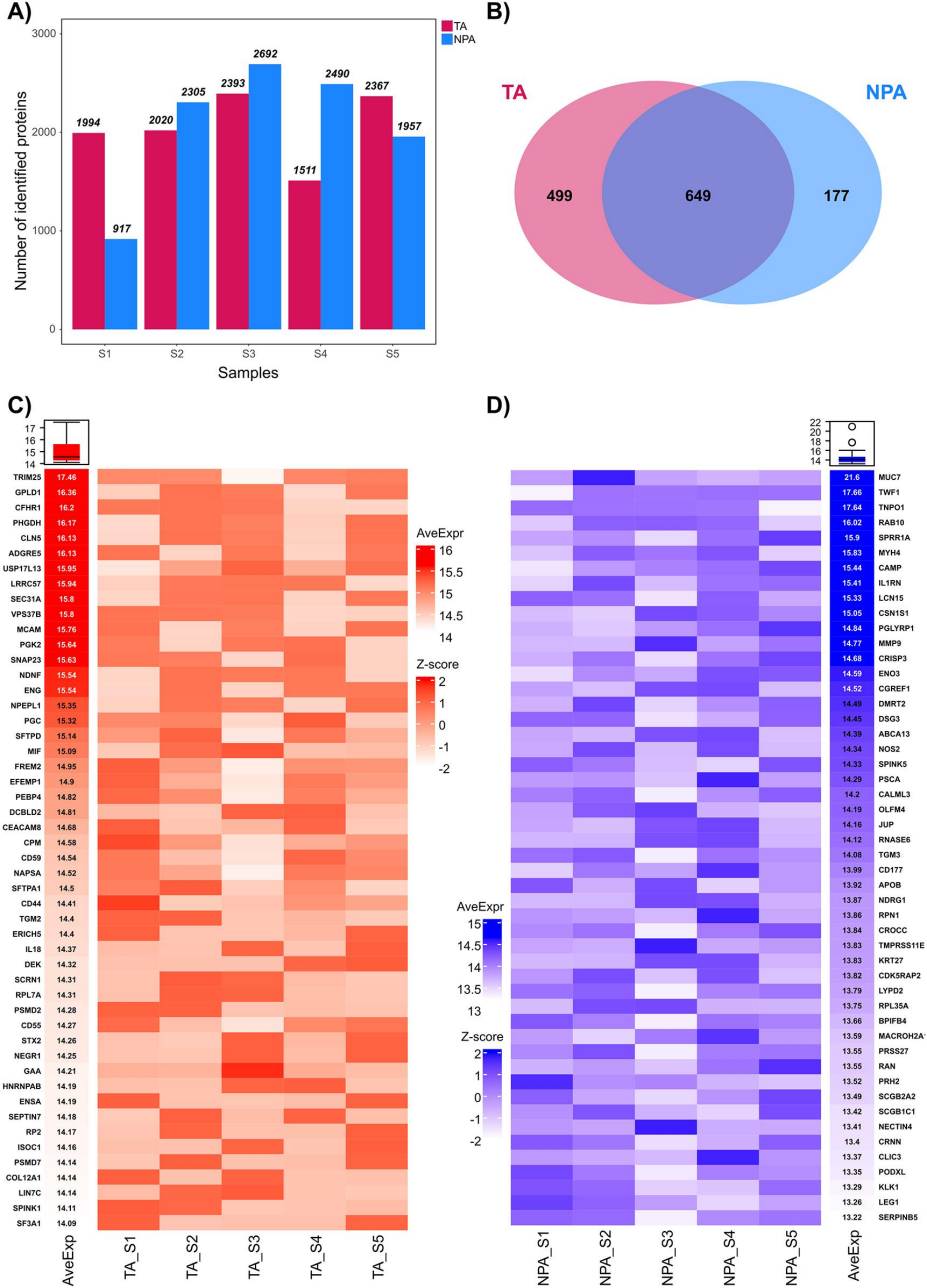
Proteome differences between tracheal aspirate (TA) and nasopharyngeal aspirate (NPA) samples. **(A)** Differences in the number of identified proteins between paired samples. **(B)** Venn diagram showing the exclusive (presence/absence) and shared proteins. **(C,D)** Heatmaps of the top 50 exclusive proteins for TA (red) and NPA (blue), respectively, according to the average expression value. The main heatmaps are colored according to the protein Z score, whereas the lateral heatmaps are colored according to the average expression value of each protein. AveExp: average expression value.

A total of 5 patients with paired samples were included (*N* = 10; Table 1), including 2 no-BPD (S1 and S2), 1 BPD patient (S3) and 2 deceased patients (S4 and S5). From the original protein matrix containing 3,699 unique proteins, three additional matrices were generated after NA filtering: NPA (826 total unique proteins), TA (1,148 total unique proteins) and shared proteins (649 total unique proteins; Figure 1B). In addition, the number of proteins in each pair of sample types was calculated (Figure 1A); the median difference in the number of proteins between sample types was 410 (IQR = 299–979). Neither NPA nor TA showed overall dominance in protein numbers; rather, the sample type with the higher protein count varied between samples. Notably, in S1 and S4, the two sample types exhibited strictly opposite count in the numbers of proteins (Figure 1A).

**Table 1:** Patient clinical characteristics.

Conditions	Overall, *N* = 5	No-BPD, *N* = 2	BPD, *N* = 1	Deceased, *N* = 2
Sex, female, %	3 (60%)	1 (50%)	1 (100%)	1 (50%)
GA (weeks)	25.2 (0.8)	26.0 (0.0)	25.0 (–)	24.5 (0.7)
Weight (g)	810.8 (200.1)	1,012.0 (31.1)	550.0 (–)	740.0 (14.1)
Days of IVM at 1 week	5.8 (2.7)	7.0 (0.0)	7.0 (–)	4.0 (4.2)
Death, %	2 (40%)	0 (0%)	0 (0%)	2 (100%)
Days of hospitalization	64 (37)	76 (5)	114 (–)	27 (1)
Respiratory modality at 1 week				
IMV	5 (100%)	2 (100%)	1 (100%)	2 (100%)
BPD grade				
No-BPD	2 (40%)	2 (100%)	0 (0%)	0 (0%)
Grade 1	0 (0%)	0 (0%)	0 (0%)	0 (0%)
Grade 2	1 (20%)	0 (0%)	1 (100%)	0 (0%)
Grade 3	0 (0%)	0 (0%)	0 (0%)	0 (0%)

GA, gestational age; BPD, bronchopulmonary dysplasia; IVM, invasive mechanical ventilation. *n* (%); Mean (SD, “–” if not possible).

### Comparative proteome analysis

A total of 177 proteins were identified as exclusive to NPA, and 499 were identified as exclusive to TA (present/absent between groups; Figure 1B). Figures 1C,D present the top 50 proteins by average abundance for each sample type, showing consistent presence across individual samples.

**Figure 2:**
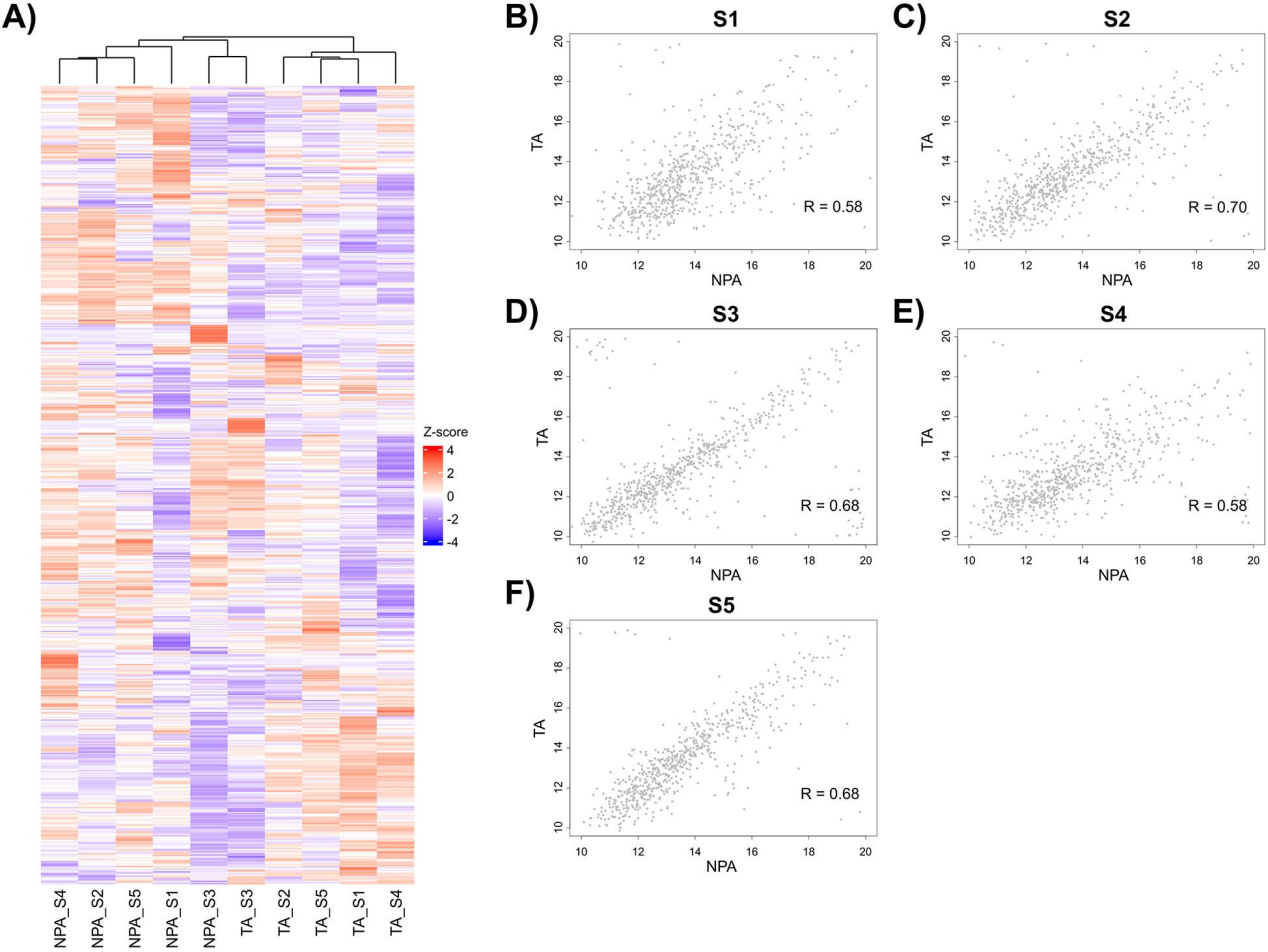
Analysis of proteins shared between tracheal aspirate (TA) and nasopharyngeal aspirate (NPA) samples. **(A)** Heatmap of all the shared proteins. Feature clustering was performed, as was sample clustering (using the “Ward D” method). The heatmap cells were colored according to the Z score. **(B–F)** Correlation plots of each paired sample. Each axis corresponds to TA or NPA sample type; R^2^ values are shown in each plot (Pearson correlation tests).

A heatmap was generated for the SP across all individual samples (Figure 2A). Sample clustering revealed differences in expression levels between SP, identifying two main clusters: one containing all NPA samples and a single TA sample and the other comprising only TA samples. Within the first cluster, S3 was the only paired sample which presented similar protein expression patterns between the NPA and TA sample types.

Pearson correlation tests were conducted to examine the correlations between paired samples (Figures 2B–F). S1 and S4 exhibited the lowest values (both 0.58, *P* value < 0.001), whereas S2 had the highest R (0.70, *P* value < 0.001), and S3 and S5 had R values close to that of S2 (both 0.68, *P* value < 0.001). Thus, nearly all samples had R coefficients above 0.60 (36% of shared variance between sample types), which indicated moderated correlation in protein expression between NPA and TA samples.

### Proteome functional enrichment analysis

#### Common terms analysis

**Figure 3:**
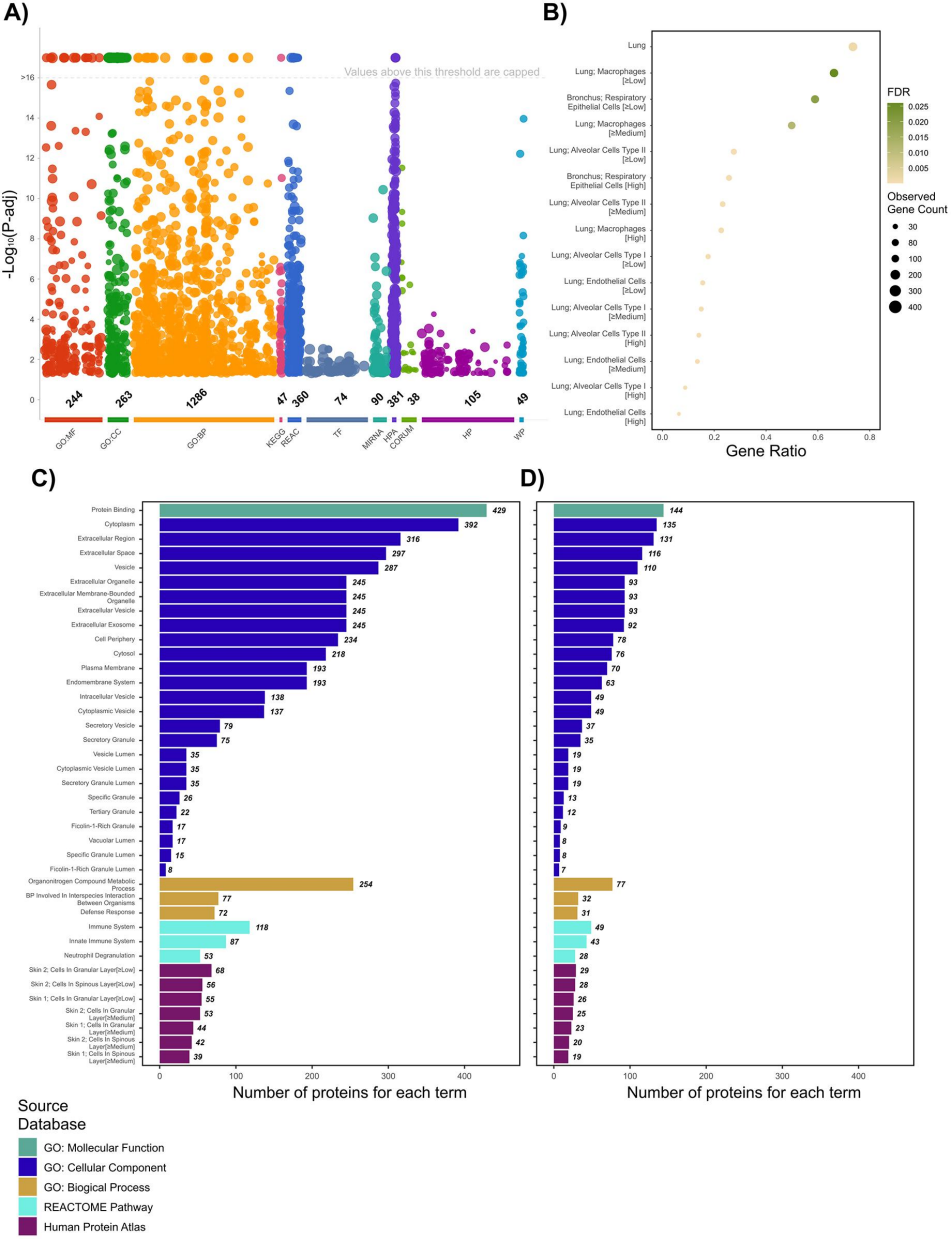
Functional enrichment analysis of common proteins and terms (functions, structures and tissues related to the studied proteins) between groups. **(A)** Enrichment results of proteins common to tracheal aspirate (TA) and nasopharyngeal aspirate (NPA) samples. The terms are ordered according to *P*-adjusted value; the colors represent the databases checked with the package gprofiler2, and the number of terms from each database is highlighted in bold. **(B)** Lung-specific common proteins common between sample types. The terms were ordered by gene ratio (the number of genes in our protein pool corresponding to a term divided by the total number of genes annotated with that term). **(C,D)** Enriched terms shared by exclusive TA and NPA proteins (present/absent in both groups), respectively. The number of proteins related to each term is shown in bold at the end of each bar. The colors represent the corresponding databases; FDR: false discovery rate.

First, human database enrichment analysis, which consists of a specific proteomic database search of functions, structures and tissues to which our proteins are related (generalized as “terms” or “enriched terms”), was applied to SP: gprofiler2 revealed a total of 2,937 significant terms (FDR < 0.05) distributed among all available databases, with potential overlap among terms (Figure 3A). Specific transcription factors (TFs) and microRNAs (miRNAs) were also included in the enrichment analysis, with 74 TF and 90 miRNA terms identified. gprofiler2 allowed TF identification by aligning TF promoters with our input using the Transfac database, while miRNAs were identified using miRTarBase, which returns a list of significantly associated miRNA for a given input. A search for HPA terms related to lung and bronchial tissue was performed to assess tissue specificity (Figure 3B). A total of 392 proteins present in our SP matrix were related to lung tissue (60% of total SP; Table 2; Figure 3B), including BPD-related lung cell types, such as alveolar type I and II cells, macrophages and endothelial cells (Table 2; Figure 3B).

**Table 2:** Lung tissue-specific enrichment results from the human protein atlas (HPA) for shared proteins from tracheal aspirate and nasopharyngeal aspirate samples.

Term Name	Database	NOG^a^	Gene Ratio	FDR^b^
Lung; Endothelial Cells [High]	HPA	34	0.063789869	6,91845E-06
Lung; Alveolar Cells Type I [High]	HPA	47	0.088180113	4,01159E-05
Lung; Endothelial Cells [ ≥ Medium]	HPA	72	0.135084428	1.81504E-10
Lung; Alveolar Cells Type II [High]	HPA	75	0.140712946	2.21416E-11
Lung; Alveolar Cells Type I [ ≥ Medium]	HPA	80	0.150093809	1.25518E-07
Lung; Endothelial Cells [ ≥ Low]	HPA	83	0.155722326	1.13227E-10
Lung; Alveolar Cells Type I [ ≥ Low]	HPA	94	0.176360225	6.56664E-08
Lung; Macrophages [High]	HPA	121	0.227016886	9.79179E-11
Lung; Alveolar Cells Type II [ ≥ Medium]	HPA	124	0.232645403	5.21027E-15
Bronchus; Respiratory Epithelial Cells [High]	HPA	137	0.257035647	2.22943E-07
Lung; Alveolar Cells Type II [ ≥ Low]	HPA	147	0.275797373	2.7488E-16
Lung; Macrophages [ ≥ Medium]	HPA	266	0.499061914	0.011669404
Bronchus; Respiratory Epithelial Cells [ ≥ Low]	HPA	314	0.589118199	0.019277793
Lung; Macrophages [ ≥ Low]	HPA	353	0.662288931	0.025966512
Lung	HPA	392	0.735459662	0.001172021

a Number of genes.

b false discovery rate.

Subsequently, we found 39 terms from different databases (GO, REACTOME, HPA) that were shared among the exclusive proteins of both sample types (Table 3; Figures 3C,D). Common functions were mainly related to immune response; cellular components varied among extracellular and intracellular vesicles, membrane and its components; and skin tissue was also enriched in both sample types. TA presented a greater number of proteins for each of the shared terms than did NPA (Figures 3C,D).

**Table 3:** Shared enrichment terms (functions, structures and tissues related to the studied proteins) between specific proteins from tracheal aspirates and nasopharyngeal aspirates.

Term Name	Database	NOG NPA^a^	NOG TA^a^
Skin 1; Cells in Spinous Layer [ ≥ Medium]	HPA	19	39
Skin 2; Cells in Spinous Layer [ ≥ Medium]	HPA	20	42
Skin 1; Cells in Granular Layer [ ≥ Medium]	HPA	23	44
Skin 2; Cells in Granular Layer [ ≥ Medium]	HPA	25	53
Skin 1; Cells in Granular Layer [ ≥ Low]	HPA	26	55
Skin 2; Cells in Spinous Layer [ ≥ Low]	HPA	28	56
Skin 2; Cells in Granular Layer [ ≥ Low]	HPA	29	68
Neutrophil Degranulation	REAC	28	53
Innate Immune System	REAC	43	87
Immune System	REAC	49	118
Defense Response	GO:BP	31	72
Biological Process Involved in Interspecies Interaction Between Organisms	GO:BP	32	77
Organonitrogen Compound Metabolic Process	GO:BP	77	254
Ficolin-1-Rich Granule Lumen	GO:CC	7	8
Specific Granule Lumen	GO:CC	8	15
Vacuolar Lumen	GO:CC	8	17
Ficolin-1-Rich Granule	GO:CC	9	17
Tertiary Granule	GO:CC	12	22
Specific Granule	GO:CC	13	26
Secretory Granule Lumen	GO:CC	19	35
Cytoplasmic Vesicle Lumen	GO:CC	19	35
Vesicle Lumen	GO:CC	19	35
Secretory Granule	GO:CC	35	75
Secretory Vesicle	GO:CC	37	79
Cytoplasmic Vesicle	GO:CC	49	137
Intracellular Vesicle	GO:CC	49	138
Endomembrane System	GO:CC	63	193
Plasma Membrane	GO:CC	70	193
Cytosol	GO:CC	76	218
Cell Periphery	GO:CC	78	234
Extracellular Exosome	GO:CC	92	245
Extracellular Vesicle	GO:CC	93	245
Extracellular Membrane-Bounded Organelle	GO:CC	93	245
Extracellular Organelle	GO:CC	93	245
Vesicle	GO:CC	110	287
Extracellular Space	GO:CC	116	297
Extracellular Region	GO:CC	131	316
Cytoplasm	GO:CC	135	392
Protein Binding	GO:MF	144	429

NPA, Nasopharyngeal aspirate; TA, Tracheal aspirate. HPA, Human Protein Atlas; REAC, REACTOME; GO, Gene Ontology; BP, Biological Process; CC, Cellular Component; MF, Molecular Function.

a Number of genes.

#### Sample-specific terms

**Figure 4:**
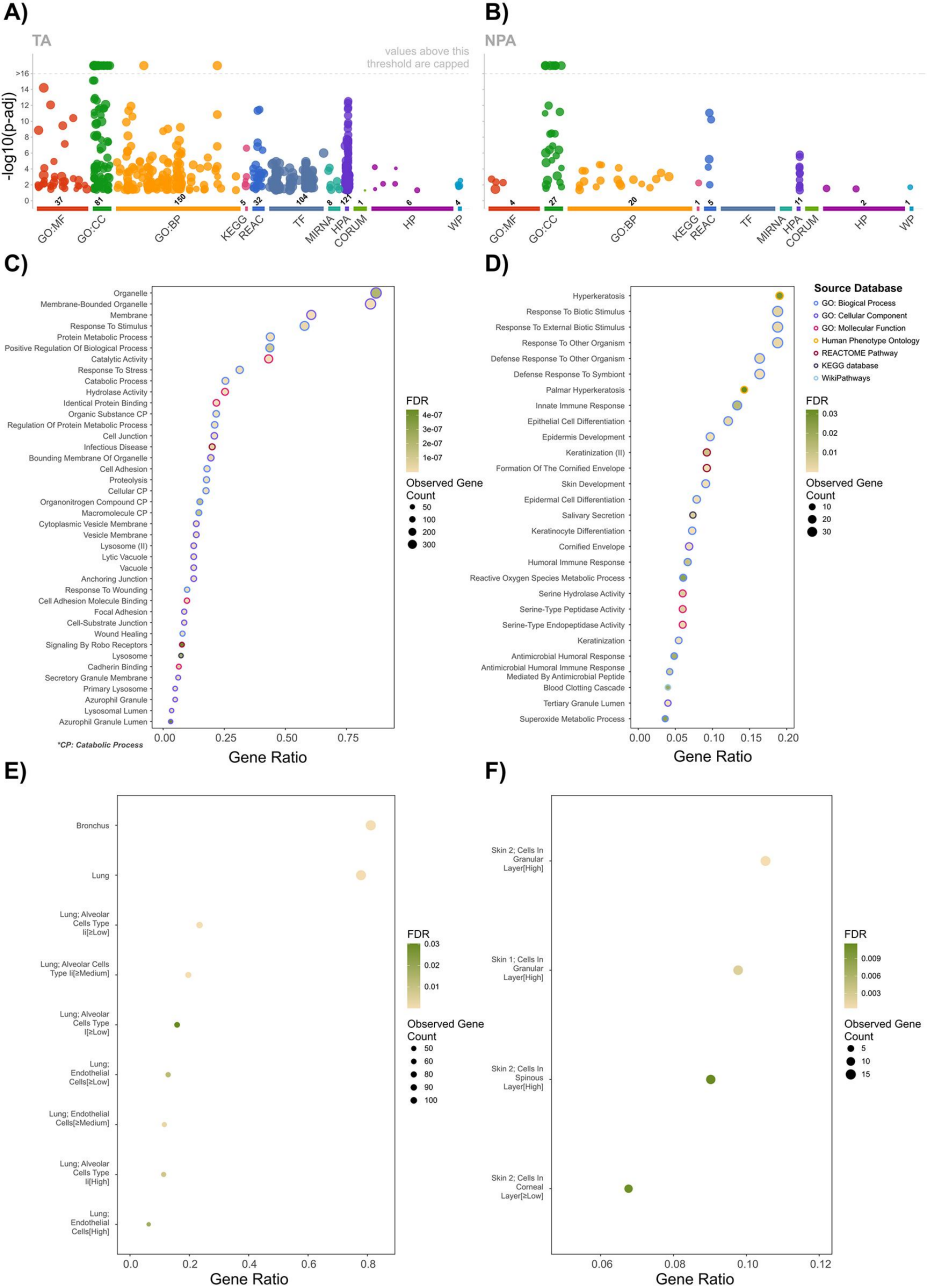
Functional enrichment analysis of exclusive proteins (presence/absence) in the tracheal aspirate (TA) and nasopharyngeal aspirate (NPA) samples. **(A,B)** Enrichment results of TA- and NPA-specific proteins, respectively. The terms (functions, structures and tissues related to the studied proteins) are ordered according to their *P*-adjusted values; the colors represent the databases searched with the gprofiler2 package; and the number of terms from each database is highlighted in bold. **(C)** The 40 terms with the highest gene ratios and lowest false discovery rates (FDRs) associated with TA-specific protein enrichment. **(D)** All specific enriched terms of NPA-specific proteins, ordered according to the gene ratio. (**E, F**) Tissue enrichment via the Human Protein Atlas of lung-specific proteins found in TA samples and NPA-specific proteins, respectively.

TA enrichment revealed 549 significant terms (FDR < 0.05); this number was reduced to 510 after eliminating shared terms. A total of 104 specific TFs and 8 miRNAs were found (Figure 4A). Figure 4C shows the top 40 terms with the lowest FDRs, ordered by gene ratio (ratio of the number of query genes or proteins to the total number of genes for each term). NPA enrichment analysis revealed 71 significant terms (FDR < 0.05), 32 of which were present only in NPA samples, and no TFs or miRNAs were found in this group (Figure 4B).

The functions of specific NPA proteins range from keratinization and epithelial development to immune response and blood clotting cascades (Figure 4D). With respect to TAs, some functions were similar to those associated with NPA enrichment but involved a wider array of metabolic processes; specific cellular component terms, such as cell adhesion complexes, lysosomes, vacuoles and organelles, were also observed (Figure 4C). TA-specific tissue enrichment (HPA) highlighted the presence of many proteins specific to lung and bronchial tissue (309 and 322, respectively; Figure 4E), as well as their alveolar and endothelial cell types. In contrast, NPA tissue enrichment via HPA identified proteins specific to skin layers, including the corneal, granular, and spinous layers (Figure 4F). Semantic similarity analysis carried out with Gene Ontology (GO) terms for each specific enrichment resulted in best match-average (BMA) similarities of 44%, 56% and 78% for biological process, molecular function and cellular component, respectively. In this regard, these metrics should be interpreted as purely descriptive, as they do not rely on statistical inference but instead provide a relative measure of functional relatedness based on GO graph topology and annotation structure.

## Discussion

With respect to our main objective, we elucidated the potential of NPA as a useful tool for research on respiratory diseases in ELGANs, given the similarities between NPA and TA samples. First, we identified 649 SP between the NPA and TA samples. Initial common protein analysis highlighted differences in protein expression but moderated correlation values. Functional enrichment analysis identified 2,937 terms, with 392 (60.4%) and 312 (48.1%) of the 649 proteins directly related to lung and bronchial tissue, respectively; in addition, a relevant number of TFs (74) and miRNAs (90) were found. Second, the TA samples exhibited greater lung tissue specificity, whereas the NPA samples were enriched in terms related to epithelial tissue. In addition, semantic similarity analysis revealed that the shared GO terms had similarities in terms of shared GO molecular functions and cellular components (56% and 78%, respectively). These findings could help clarify the application of NISM for monitoring the lung health of ELGANs, particularly in scenarios where ISMs are not strictly necessary.

Shared protein enrichment revealed specific TFs and miRNAs. TFs are highly specific molecules that regulate gene expression and processes such as cell development and differentiation (31). Although they are rarely detected in proteomic studies due to their tendency to bind to other molecules or become activated under specific stimuli (31), enrichment-based approaches allow the inference of their potential regulatory roles. On the other hand, miRNAs constitute a family of small noncoding RNAs with a strong influence on the regulation of gene expression (7, 32, 33); in addition, their expression has been linked to specific tissues and developmental stages (33). Several studies have linked miRNAs to lung pathologies including cancer (32, 33) and lung inflammatory diseases (7, 34). Harrel et al. (35) recently reviewed the roles of miRNAs in the regulation of pulmonary fibrosis, a process that is shared by pathologies including pulmonary hypertension and BPD; furthermore, these authors reviewed the roles of miRNAs as promoters of lung inflammation via the promotion of excessive fibroblast differentiation. Overall, shared TFs and miRNAs between NPA and TA were identified; nevertheless, these findings may reflect relative specificity of NPA with respect to pulmonary tissue and pathology.

One of the most important characteristics of BPD is pulmonary simplification, a process that is strongly influenced by the disruption of lung tissue development caused by prematurity (2, 36). More specifically, alveolar cell simplification has been identified as one of the main factors in BPD (37–39). Research on alveolar cells has been carried out with respect to miRNA (40) and endothelial progenitor cells (38), and several studies have investigated the roles of specific antioxidant molecules, such as nuclear factor erythroid 2 like 2 (39). This research has emphasized the crucial roles of lung vascularization, reactive oxygen species regulation and inflammation. Notably, more than 60% of the SP identified here were related to lung and bronchoalveolar tissues. Thus, this specificity is of high interest regarding lung and BPD-related pathologies.

However, compared with the TA samples, the NPA samples lacked lung-specific proteins, as well as TFs or miRNAs. TA samples exhibited a greater number of exclusive proteins (499), including a greater number of lung-specific proteins and representing a wider range of enriched terms. This was also evident in the GO biological process similarity of the two types of samples (44%). In contrast, both sample types had distinct proteins involved in shared processes and tissues. Some of these shared terms, such as immune system or neutrophil degranulation, have been linked to BPD (12); furthermore, the lack of epithelial development (41) and the recruitment of intra- and extracellular membrane proteins (2) are factors related to this disease. These results reveal differences in sample collection regions, reflecting that protein expression patterns may vary throughout the respiratory airway system.

Although we have highlighted the useful characteristics of NPA as a NISM for future BPD studies, our study also has several limitations. With respect to sample size, the lack of TA sample availability has limited our study resolution and power. TA requires previous patient intubation (7), a practice that has been reduced substantially at NICUs throughout the past decade due to its implications for patient health (2, 9). Another related limitation is the relatively small number of patients in each studied group (no-BPD, BPD and deceased). This has clearly reduced our ability to assess potential differences between groups, which could reduce the resolution of our study.

With respect to sample limitations, we studied the potential of NPA as a relatively easier sample collection methodology, which could dramatically increase patient recruitment for studies of lung biomarkers in preterm neonates (21) relative to the availability of TA samples. On the other hand, we observed differences in the patterns of SP between NPA and TA patients and between no-BPD and deceased patients but did not observe such a pattern in our BPD patient. This patient's shared proteome pattern was consistent between the NPA and TA samples, which suggests that BPD induces changes in the expression patterns of proteins throughout the respiratory airway system. However, given the small sample size, the qualitative nature of our study, and the potential heterogeneity in protein expression across patients, including differences in clinical conditions between no-BPD, BPD and deceased groups, this issue needs further exploration studies in the future. Thus, we expect to increase the sample size through collaboration with other clinical research centers and NICUs. In addition, the presence/absence logic may be influenced by the detection variability inherent to proteomic analyses; in this regard, “exclusive” proteins may not always reflect true biological absence. Accordingly, this analysis emphasizes consistently detected proteins across samples, prioritizing robustness and interpretability. Finally, enrichment analyses were approached on gene IDs derived from the identified proteins, which may not fully reflect protein-level regulation. To this matter, the TFs and miRNAs identified should be interpreted as putative upstream regulators inferred from enrichment analysis, rather than directly measured entities.

Significant efforts have been made to establish NISMs as tools for respiratory disease monitoring in ELGANs (16, 17, 42, 43). Su et al. (43) carried out targeted cytokine assays to search for early BPD biomarkers. Saima et al. revealed the potential of early biomarkers in the urine (16) and oral secretion (17) proteomes of preterm infants. Specifically, urine proteomics revealed the discriminative potential of a combined panel of chitinase-3-like protein-1 and frizzled-6 [AUC = 0.92; 95% CI (0.84–1.00)] (16), and 10 differentially expressed proteins in oral secretions were highly correlated with BPD (17). These results, similar to our findings (21), highlight the potential capability of NISM proteomics. Nevertheless, the discriminative ability of these respiratory proteomic biomarkers coupled with other early monitoring assays for ELGANs remains unknown. Our group has demonstrated the potential of lung ultrasound as a great tool for the assessment of BPD and other respiratory comorbidities at 3–7 days of life (44). Thus, various NISMs may become more useful for proteomic and clinical variable studies in the coming years.

To conclude, our findings reveal proteomic similarities between NPA, a NISM, and TA, a broadly used ISM. High-throughput proteomic samples from both sample types presented moderated correlation values. More than 60% of the SP from both the NPA and TA samples were specifically related to lung tissues. Furthermore, enrichment analysis of the SP yielded 2,937 significant terms representing a wide variety of molecular functions, processes and specific patterns, and TFs and miRNAs were also identified. We hope that our findings will promote future improvements and the use of NPA as a useful tool for studies of BPD and other lung pathologies. Although the present work was a qualitative study with a small number of paired samples, we aim to keep using NPA as a tool for respiratory research with larger cohorts to demonstrate its potential in the near future.

## Data Availability

The original contributions presented in the study are publicly available. This data can be found here: https://github.com/fergardm/FGM-AAO-BPD.
